# A novel method for the normalization of microRNA RT-PCR data

**DOI:** 10.1186/1755-8794-6-S1-S14

**Published:** 2013-01-23

**Authors:** Rehman Qureshi, Ahmet Sacan

**Affiliations:** 1 Center for integrated Bioinformatics, School of Biomedical Engineering, Science and Health System, Drexel University, 3120 Market Street, Philadelphia, PA 19104, USA

**Keywords:** microRNA, RT-PCR, Normalization, Microarray

## Abstract

**Background:**

MicroRNAs (miRNAs) are short non-coding RNA molecules that regulate mRNA transcript levels and translation. Deregulation of microRNAs is indicated in a number of diseases and microRNAs are seen as a promising target for biomarker identification and drug development. miRNA expression is commonly measured by microarray or real-time polymerase chain reaction (RT-PCR). The findings of RT-PCR data are highly dependent on the normalization techniques used during preprocessing of the Cycle Threshold readings from RT-PCR. Some of the commonly used endogenous controls themselves have been discovered to be differentially expressed in various conditions such as cancer, making them inappropriate internal controls.

**Methods:**

We demonstrate that RT-PCR data contains a systematic bias resulting in large variations in the Cycle Threshold (CT) values of the low-abundant miRNA samples. We propose a new data normalization method that considers all available microRNAs as endogenous controls. A weighted normalization approach is utilized to allow contribution from all microRNAs, weighted by their empirical stability.

**Results:**

The systematic bias in RT-PCR data is illustrated on a microRNA dataset obtained from primary cutaneous melanocytic neoplasms. We show that through a single control parameter, this method is able to emulate other commonly used normalization methods and thus provides a more general approach. We explore the consistency of RT-PCR expression data with microarray expression by utilizing a dataset where both RT-PCR and microarray profiling data is available for the same miRNA samples.

**Conclusions:**

A weighted normalization method allows the contribution of all of the miRNAs, whether they are highly abundant or have low expression levels. Our findings further suggest that the normalization of a particular miRNA should rely on only miRNAs that have comparable expression levels.

## Background

MicroRNAs (miRNAs) are short non-coding RNA sequences that average 22 nucleotides in length [1-3]. This class of RNAs is distinct from other short sequence RNA types such as siRNA and snRNA. The first RNA of this class was identified in C. Elegans in 1993 [4]. However, miRNAs were not recognized as a special class of RNAs until a decade ago [5]. To date, all animal and plant species have been found to express miRNAs [6]. At this time approximately 1000 miRNA sequences have been identified in the human microribonucleome [7]. miRNA sequences are highly evolutionarily conserved among mammals [4,8-12]. Approximately 80% of known miRNA genes are found in intronic regions of the genome [13,14]. miRNAs are involved in many biological processes by influencing the regulation of specific target genes, generally resulting in the down-regulation of those target genes. There are two postulated methods by which miRNAs act on their target genes. If the miRNA binds with an mRNA transcript and they exhibit high complementarity, it will cause the degradation of the mRNA. If the miRNA binds with incomplete complementarity then it causes translational repression of the mRNA. In plants the primary mechanism of action of miRNAs is mRNA transcript degradation, while in animals, translational repression is more common [6]. An estimated 60% of mammalian mRNAs are targeted by one or more miRNAs [10,12].

miRNAs have been discovered to play a role in many diseases and pathologies [2,10,13,15,16]. The role of miRNAs in cancer has been examined and several miRNAs have been found to regulate tumor-related genes [1-3,10,13,17-19]. In fact, more than half of all miRNA genes are located in cancer-associated regions of the genome or in fragile sites [3,13]. As a result, therapeutic applications of miRNAs are being investigated. Furthermore, due to the link between many miRNAs and cancer, these RNA molecules are being investigated as potential cancer biomarkers. The fact that some miRNAs can be found extracellularly and maintain their stability in the extracellular environment facilitates their usage as biomarkers [10].

There are two main tools used to quantify the expression of miRNAs: microarrays and real-time polymerase chain reaction (RT-PCR). RT-PCR returns the number of cycles that the samples underwent before they were detected, reported as a value known as the Cycle Threshold (CT). The CT values vary logarithmically with expression levels. There are several methods of normalizing the data and calculating the fold-change of each gene between samples. For convenience, in this presentation the terms, "miRNA" and "gene," are used interchangeably in the context of RT-PCR. ΔCT values are calculated by subtracting the CT value of the endogenous control for a given sample (or the mean of the CT values of the endogenous controls if more than one exist) from the CT value of the gene for the given sample. In the calculation of ΔCT values we refer to the number subtracted from the raw CT values of each gene as the *CT_0_*. The ΔΔCT is calculated by subtracting the ΔCT of an experimental sample from a control sample. Fold change is calculated by raising 2 to the power of the negative ΔΔCT value, since CT values are related to the amount of miRNA or gene logarithmically [20]. The relationship between CT, ΔCT, ΔΔCT, and Fold Change (FC) are given by the equations below.

(1) $$\text{Δ}CT=CT-CT_{0}$$

(2) $$\Delta \Delta CT=\Delta CT-\Delta CT_{control}$$

(3) $$FC=2^{-\Delta \Delta CT}$$

Theoretically, endogenous controls are selected because they have low variance in their expression levels across samples. In the case of miRNAs, the endogenous controls are typically recommended by the manufacturer of the miRNA kit used in the PCR. Some of the most commonly used endogenous controls are RNU44, RNU48, and U6 [17]. However, the usage of these endogenous controls is problematic, because even though these endogenous controls have stable expression levels in normal tissue samples, they have been found to be differentially expressed in cancerous tissue compared with normal tissue [20].

Directly applying this method can lead to misleading results if the CT values in the data are not normalized. There are several commonly used methods for miRNA normalization, including: quantile normalization, median normalization, and cyclic loess. Quantile normalization involves sorting the expression values of each gene in a given sample in order from least to greatest. This is done for each sample in the study. The vectors of the sorted CT values for each sample are combined into a matrix. The mean of each row of the matrix is calculated. The CT value in each element in each row is replaced with the mean of the entire row. In the case of median quantile normalization the median of the row is used instead of the mean. The CT values in each sample are then rearranged back into their original order. This causes the distribution of CT values across all samples to assume the same shape, which will minimize the variance except for that resulting from the experimental condition beings studied [21,22].

Median normalization shifts the CT values in each sample such that the median CT value of each sample is the same. The median of each plate should be determined, and the medians of all plates should be arranged in a vector and sorted to determine the median of the medians. In each plate the difference between the median of the sample and the overall median should be subtracted from the CT value of each gene [9].

In cyclic loess normalization, pairs of plates are considered. For all pairs of plates the difference of the log of the CT for each gene is represented by *M*, and the average of each gene of the log of the expression values is represented by *A*. Then a loess curve is fit by regression of *M *on *A *which results in a fitting vector *F*. The genes in the first sample are adjusted by adding half the *F *value corresponding to the log of the CT for each gene. In the second sample half the *F *value is subtracted from the log CT of the gene [9,21].

A number of normalization methods developed for microarrays have been applied to RT-PCR experiments. These methods assume that all miRNAs present in the organism are being profiled in the experiment. While microarrays can profile all miRNAs encoded in a genome, this assumption does not hold for RT-PCR experiments which typically only profile a few hundred miRNAs at a given time [23]. Mar et al. investigated the use of quantile normalization as well as rank-invariant set normalization [24]. In rank-invariant set normalization genes are ranked by their expression for each sample and the ranked list is compared to the ranked list of genes for a reference sample. Genes are considered to be rank-invariant if they have similar ranks in the reference sample and the experimental sample. All experimental samples are compared to the reference sample and an intersection of these lists is used to identify the rank-invariant genes which can then be used for normalization [24]. Deo et al. compared several normalization methods and concluded that data-driven methods performed best. They compared normalization by endogenous controls, using the mean as a pseudo-control, and two different methods of quantile normalization. They concluded that quantile normalization performed the best despite the fact that using the mean as an endogenous control produced lower standard deviation [23].

One of the main problems with RT-PCR that remains as yet unaddressed by current normalization methods is the systematic bias present within the data. We observe that standard deviation increases as CT values increase. We believe that the most likely cause of this observation is the assumption that the PCR magnification at each cycle is an exact doubling of the expression levels is inaccurate. There seems to be an accumulation of an expression-level specific rate-limiting effect. As a result, a small difference in the size of the initial sample being amplified causes larger variations in the CT values of the less abundant microRNA molecules. Consequently, using endogenous controls, which are usually chosen from highly expressed microRNAs, for normalization becomes inappropriate for the less-abundant microRNAs. Even quantile normalization has been observed to produce more variance at high CT values than was present in the original raw data [24]. One potential solution is to use the mean expression values of all genes in a sample as the endogenous control, as proposed by Mestdagh et al. [15], and calculate ΔCT by subtracting this mean CT value from the CT value of all genes in the sample. However, this approach is not ideal because the mean of the entire sample is sensitive to fluctuating genes as well as undetected genes which have high CT values. As a result, the mean-value normalization method is dominated by the large fluctuations of the less-abundant microRNAs and may cause spurious differential expression levels for otherwise stable microRNAs. In this study, we propose a method of using a weighted mean as an artificial endogenous control to calculate ΔCT values. The standard deviation of a microRNA across all samples is considered as a stability measure and each microRNA is weighted by its stability to generate the artificial endogenous control levels.

## Methods

The primary dataset used in this study was obtained from a recently deposited microRNA RT-PCR dataset in the Gene Expression Omnibus (GEO) [25]. The data was from a study by Jukic et al. that examined the difference in miRNA expression profiles in melanocytic neoplasms between young and older adults [1]. Their study examined 10 young adults and 10 older adults and measured the expression of 666 microRNAs. We used the raw CT values measured in their data to compare different approaches to normalizing the data. This dataset has been previously used by Deo et al. to compare various normalization techniques; this dataset is highly suited to the comparison of normalization studies due to the large number of samples and the use of multiple cards [23].

We have investigated several normalization methods, including quantile, mean, and median normalization methods, and endogenous controls identified using various stability criteria. In mean and median normalization, the mean and median of all of the genes in a given sample are used as the value for *CT_0_*. For identification of endogenous controls, we calculate the standard deviation of each microRNA across all samples, and rank them in the order of increasing standard deviation. The CT values of the top-*k *microRNAs are averaged in each sample to provide the *CT_0 _*values.

A new weighted mean metric is proposed using the standard deviations of the microRNAs as weights. For a given gene, the weighted average is calculated using the following equation:

(4) $$CT_{0}=\overset{n}{\underset{j}{\sum }}CT_{j}\times \frac{{\left(\frac{1}{STD\left(CT_{j}\right)}\right)}^{wmp}}{\sum_{i=1}^{n}1/STD\left(CTi\right)}$$

where *wmp *is the weighted mean power, which can be adjusted to shift the dominance between stable and unstable microRNAs, *n *is the number of genes or microRNAs, and STD is a function that returns the standard deviation. The weighted mean calculation involves raising the inverse of the standard deviation of a given gene across all samples to the weighted mean power, which is usually specified as 1, and dividing by the sum of the inverses of the standard deviations for all genes. *CT_0 _*is calculated for each sample by taking the sum of the product of all the raw CT values in the sample and the previous number. When the ΔCT is calculated the CT of each gene is subtracted by the above value. This method gives a higher weight to genes with a lower standard deviation.

We also examined the reproducibility of miRNA expression experiments between RT-PCR and microarray. To explore this topic we utilized data from Chen et al. [26]. They evaluated miRNA expression in murine myoblasts utilizing both RT-PCR and microarrays. They evaluated the consistency of different RNA preparation methods for RT-PCR. We harnessed their data to explore the correlation of RT-PCR with microarray. We further explored whether the expression level of a particular miRNA in RT-PCR would bias its correlation with its expression on a microarray.

## Results and Discussion

**Figure 1:**
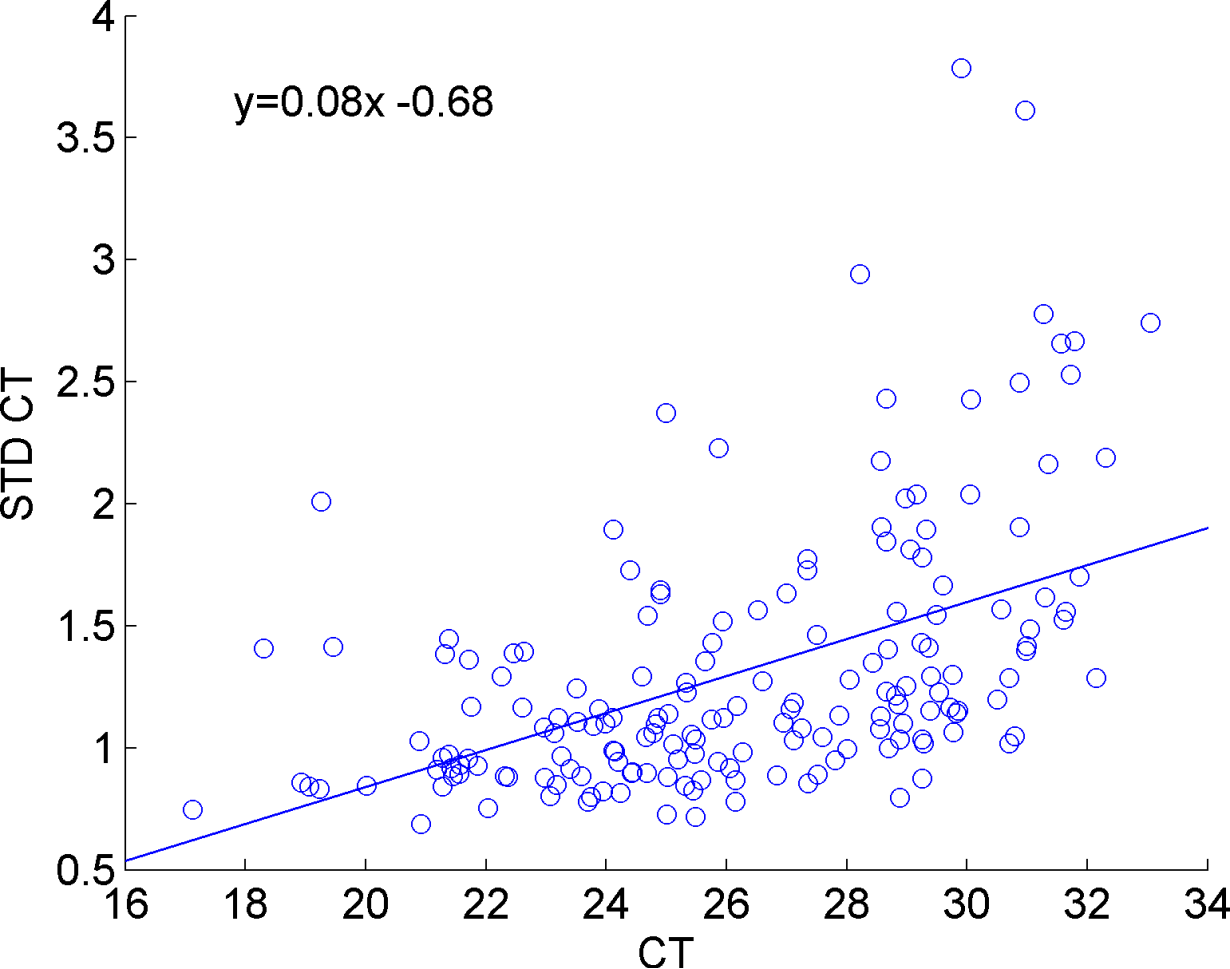
**Dependence of variability on expression level**. Each point represents the standard deviation versus the mean of the CT values for a particular microRNA across all samples.

In order to test the hypothesis that increasing CT values magnifies the natural variation between the initial amounts of samples loaded in each well during RT-PCR, we examined the standard deviation of the genes against their mean CT values, as shown in Figure 1. The application of linear regression to this data clearly shows a trend of increasing standard deviation values for higher CT values. Note that the higher the CT value, the more cycles were required to observe that microRNA, hence the less abundant that microRNA was in the initial loaded sample.

**Figure 2:**
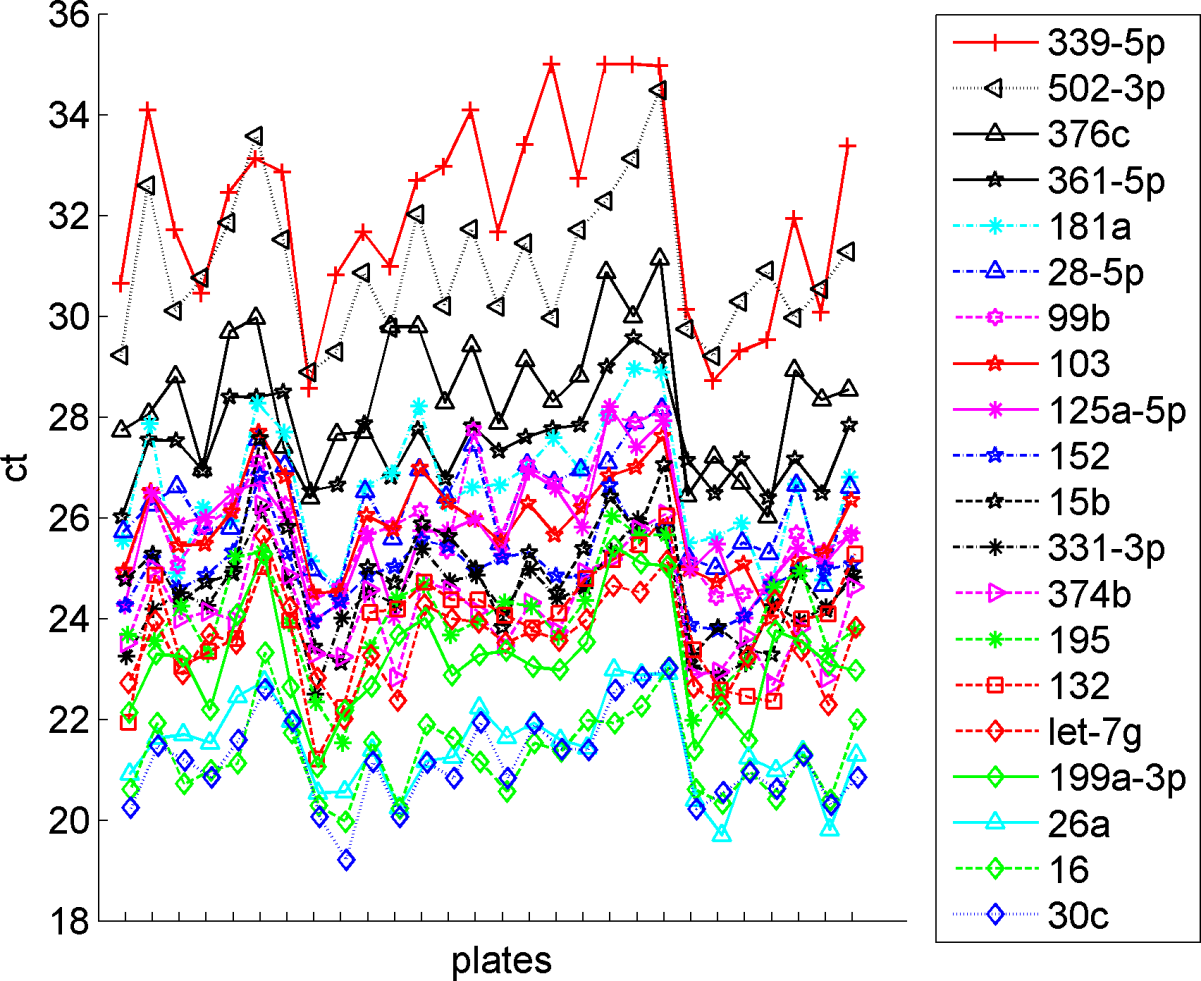
**miRNAs most correlated with the mean expression value**. The CT values of 20 miRNAs where the change between samples is most correlated with the change in the mean expression value from sample to sample.

As expected, the CT values of most genes are well correlated with the mean expression of all the genes. This is illustrated in Figure 2, where we show the expression of the 20 miRNAs that are most correlated with the mean expression. Each tick on the x-axis represents a unique experimental sample.

**Figure 3:**
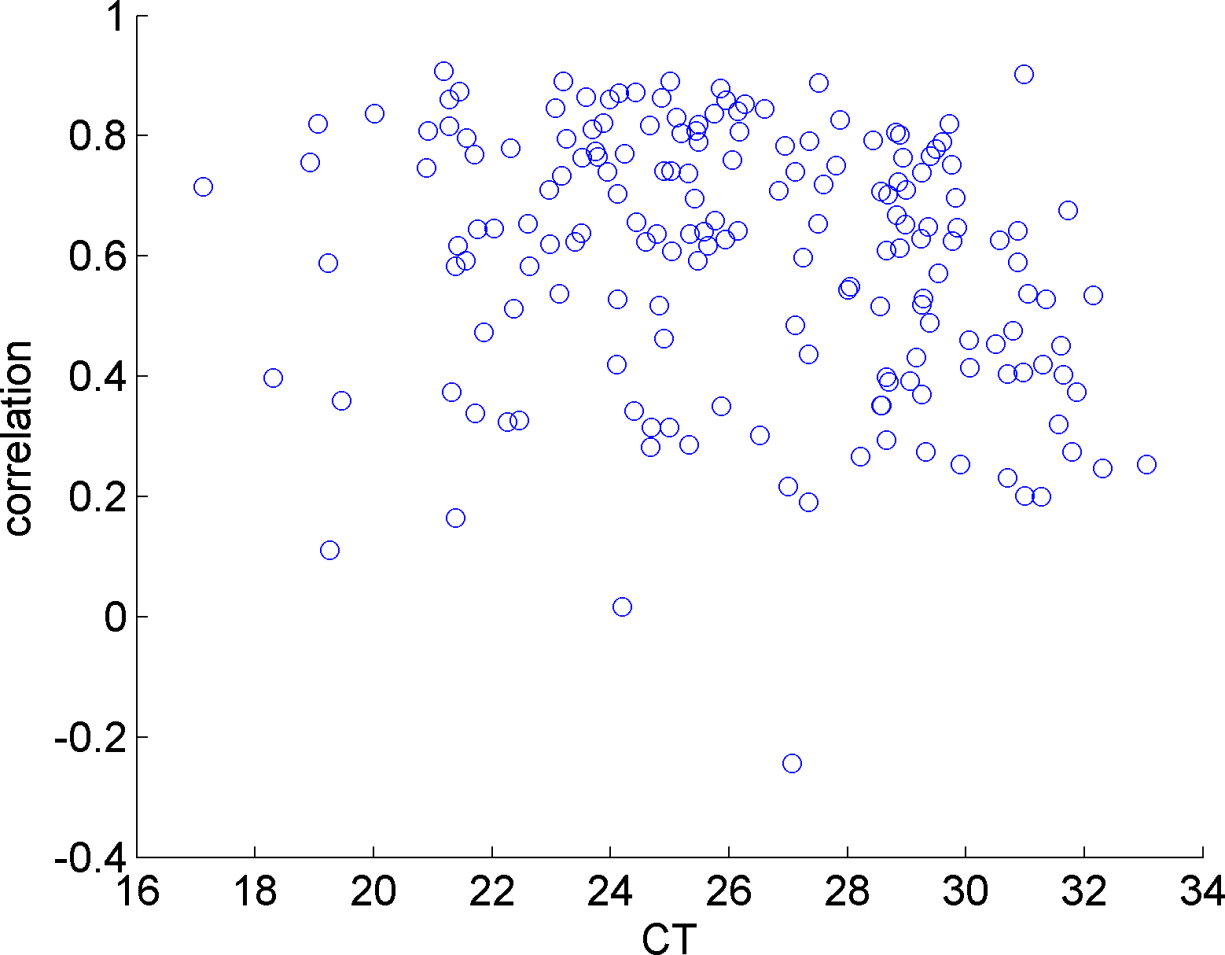
**Correlation with mean vs. mean CT value**. Each point represents the correlation of a particular miRNA with the mean expression of all miRNAs across multiple samples vs. the mean of the CT value of that particular microRNA. The x-values are the mean CT of a miRNA, and the y-values are the correlation of the vector of sample differences from the mean for each miRNA with the vector of the sample mean differences from the overall mean.

The correlation with the mean expression level extends to low-abundant miRNAs. We demonstrate this in Figure 3, wherein the Pearson correlation coefficient of the fluctuations in each gene with respect to its own average is shown against the fluctuations of the mean expression levels of all genes. The plot shows that a high correlation is observed whether the mean CT values are low or high.

**Figure 4:**
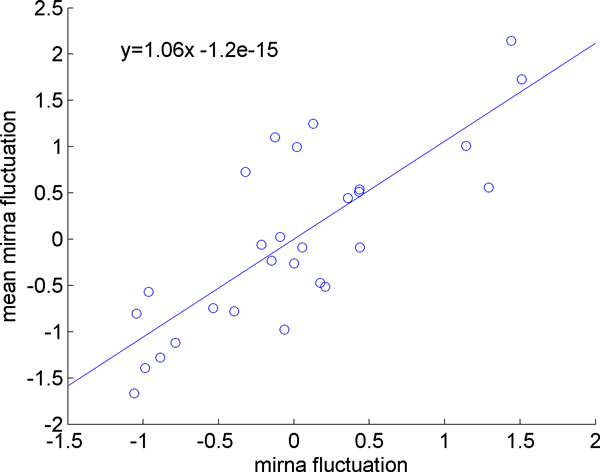
**Correlation of miRNA fluctuation with mean fluctuation**. Each point represents a single sample in the study. The x-value of the point is the difference of the sample mean from the overall mean and the y-value is the difference between the expression of an miRNA in that sample and the mean expression of that miRNA across all samples. The slope of the line quantifies the sensitivity of fluctuations in the miRNA's value to fluctuations in the overall mean. This plot is presented as an example for a single microRNA; all miRNAs were plotted in this fashion.

**Figure 5:**
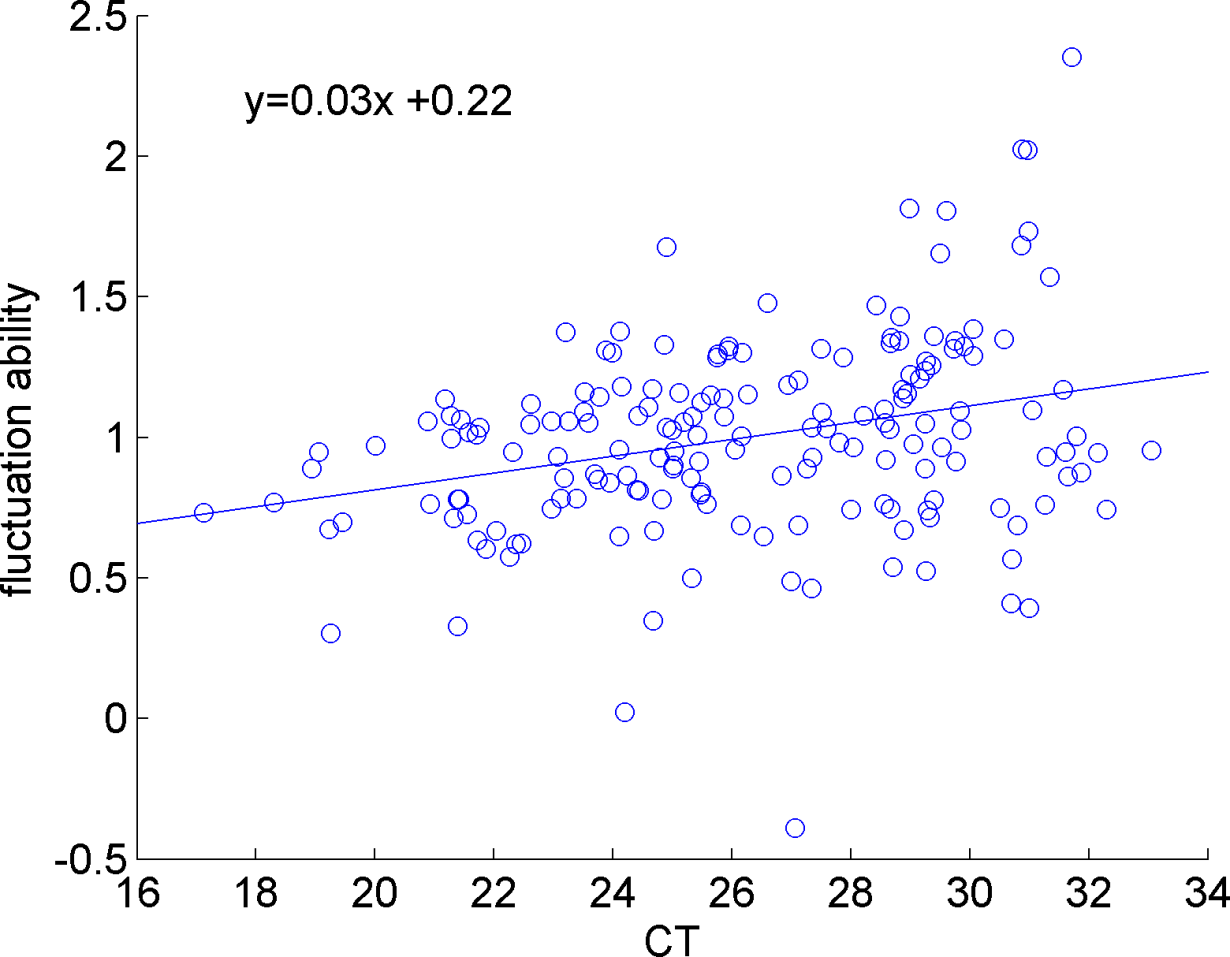
**A plot of fluctuation response vs. expression level**. Each point represents the slope of a particular miRNA as shown by example in Figure 4. All miRNAs' slopes are plotted against their mean CT to show that as CT increases the response to sample fluctuations also increases.

**Figure 6:**
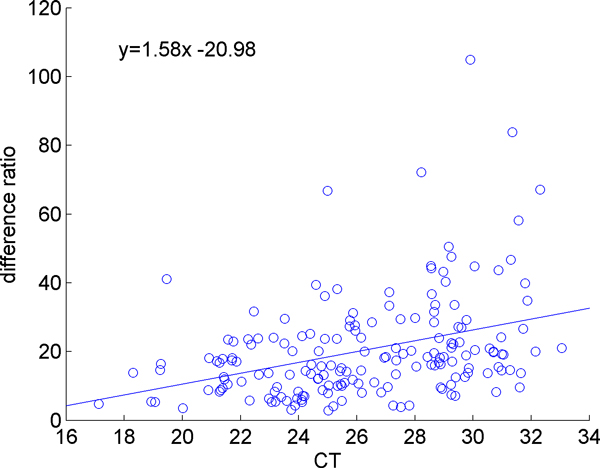
**Difference ratio vs. expression level**. Each point represents the ratio of the differences in expression level of a microRNA and the mean of all microRNAs against the mean of the CT values for that particular microRNA across all samples. The difference ratio is calculated by dividing the difference of a miRNA's expression in a particular sample by the difference between that sample's mean and the overall mean. For each miRNA a vector of difference ratios is calculated with one value for each sample. On the figure the y-axis represents the mean difference ratio for a particular miRNA. The ratio of this difference increases with increasing CT, demonstrating that lowly expressed miRNAs are more sensitive to fluctuations in the mean.

In order to quantify the sensitivity of the microRNA expression levels to the initial loaded sample size, a regression line is fitted to the fluctuation of each miRNA against the fluctuation of mean expression. Fluctuation is determined by subtracting the value of the expression of a miRNA in a given sample from the mean expression of that miRNA for all samples; the overall mean of the expression of all miRNAs in all samples can be subtracted from the sample means to determine the fluctuation of a sample mean. In Figure 4 we demonstrated this for a single miRNA. The x-value of each point is the difference between a sample mean and the overall mean, and the y-value is the difference between the expression of that particular miRNA in that sample and the mean expression of that miRNA across all samples. The slope of the line indicates how sensitive the miRNA is to initial sample size, with larger slope values corresponding to larger variations in response to a small change in sample size. The slopes were calculated for all miRNAs. Figure 5 shows the sensitivity of each miRNA, which is determined by the slope calculation demonstrated in Figure 4, versus against the overall mean expression level of that miRNA. We observe that the sensitivity is expression level dependent. Highly expressed miRNAs (those with small CT values) are less responsive to changes in the overall mean of the miRNAs, whereas the low-abundant genes are more sensitive to the changes in the overall mean of the miRNAs. Note that, this is not simply a random effect due to low abundant microRNAs being more variable, since the variation is still correlated and is in the same direction of the change in mean expression level. The same observation is made by examining the ratio of the fluctuations in individual miRNAs to the fluctuations of the mean expression level, as shown in Figure 6.

In conclusion, the fluctuations of the low-abundant miRNAs are not random. The changes in their expression levels are correlated well with the overall changes in all miRNAs, which is assumed to be due to different starting sample sizes for the PCR reactions. We see that there is a systematic bias in the CT values that causes the expression levels of the low-abundant miRNAs to be more sensitive to the initial sample sizes.

**Figure 7:**
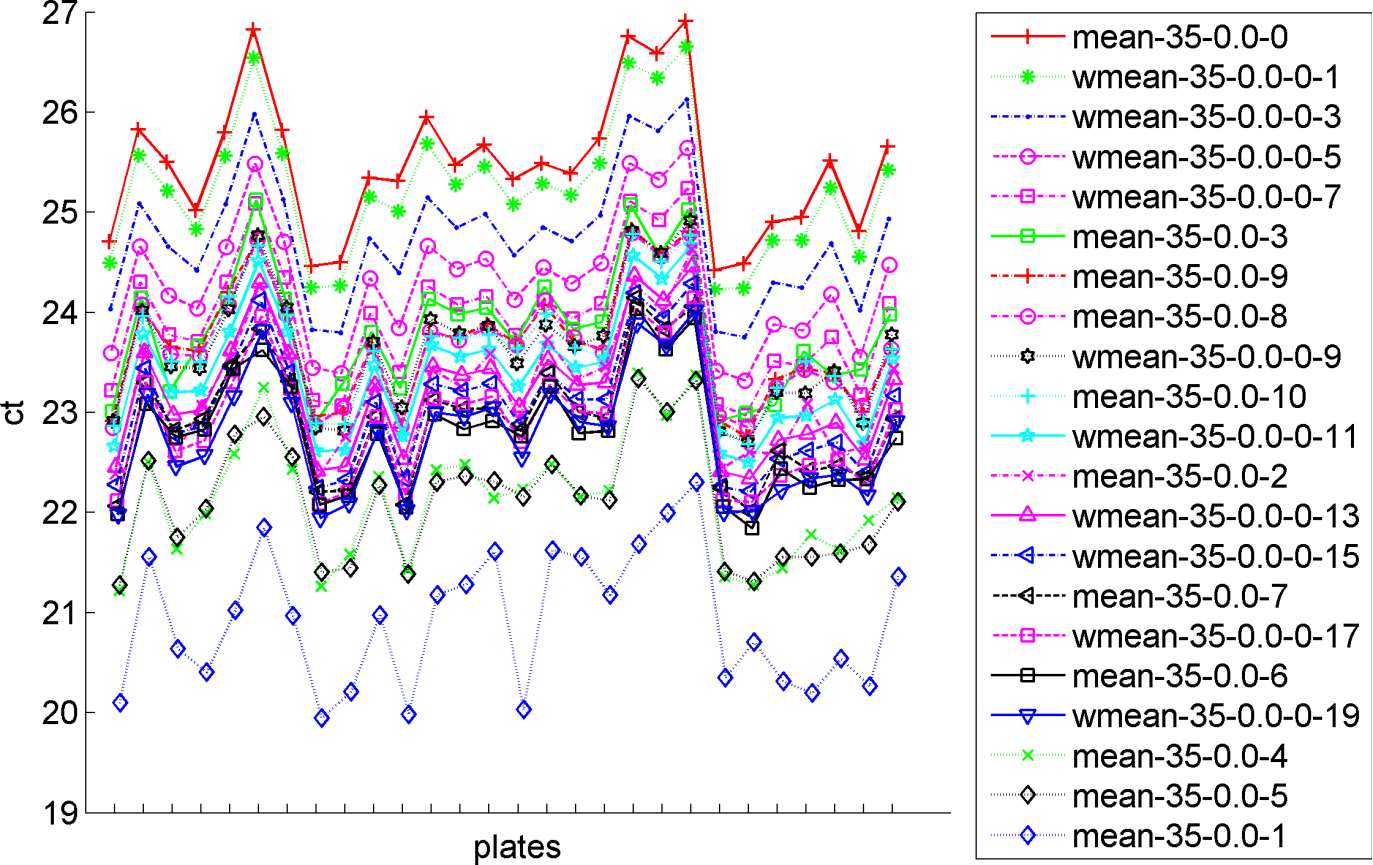
**Comparison of the *CT_0 _*values generated by different normalization methods**. The *CT_0 _*calculations of each normalization method for each sample are shown here. The legend indicates the method used. The last number on the legend shows either the *k *value in a top-k calculation for the mean normalizations or the weighted mean power for the weighted mean normalizations.

We then investigated the suitability of our weighted mean metric. In Figure 7 we display the values for *CT_0 _*for several different methods including using the mean of all raw CT values in the bottom line (top-*k *= 0), the means of the top-*k *miRNAs for different values of *k*, and the weighted mean for different values for the weighted mean power. The plot demonstrates that varying the weighted mean power enables the shifting of the curve upwards or downwards. In Table 1 and Table 2, we compare the resulting means, standard deviations, and geNorm stability values [27] for mean and weighted mean normalizations, respectively. We repeat analysis this for the top 10 genes, with the lowest standard deviation in 3. We see slightly higher standard deviations in the weighted mean normalization method compared to the top-*k *calculations, but the weighted means' *CT_0 _*values are determined to be more stable by geNorm (the lower the value the more stable). In Table 3, we see that the best individual miRNAs have a much higher standard deviation and are much less stable than any of the *CT_0 _*calculations using either the top-*k *miRNAs or the weighted mean. This indicates that it is better to use these values in the ΔΔCT calculation than any endogenous control. We also investigated the use of the geometric mean of the endogenous controls, all of the miRNAs, or just the top-*k *miRNAs. In all cases using the geometric mean resulted in similar but slightly lower values for CT_0 _as using the mean and almost exactly the same standard deviations and geNorm stability values (data not shown), thus using the geometric mean had no advantage over using the mean.

**Table 1:** Mean Normalization

top-k	mean *CT_0_*	stdev	geNorm
1	20.92	0.69	0.35
2	23.2	0.64	0.21
3	23.8	0.64	0.19
4	22.13	0.63	0.2
5	22.11	0.61	0.17
6	22.79	0.6	0.18
7	22.91	0.61	0.16
8	23.66	**0.59**	**0.15**
9	23.67	0.6	0.16
10	23.61	0.61	0.16
∞	25.59	0.71	0.23

Mean *CT_0 _*calculations, standard deviations, and geNorm stability values for using the mean of the top-k miRNAs with lowest standard deviation as the *CT_0 _*value. An infinite value for k signifies the mean of all miRNAs. The best values are marked in bold.

**Table 2:** Weighted Mean Normalization

power	mean *CT_0_*	stdev	geNorm
**1**	25.34	0.69	0.21
**3**	24.82	0.67	0.18
**5**	24.35	0.65	0.15
**7**	23.96	0.64	0.14
**9**	23.65	0.63	0.13
**11**	23.41	0.62	0.12
**13**	23.21	0.62	0.12
**15**	23.04	0.62	0.12
**17**	22.89	0.61	0.12
**19**	22.76	0.61	0.13

Mean *CT_0 _*calculations, standard deviations, and geNorm stability values for weighted mean normalization using different values of the weighted mean power.

**Table 3:** Using the top 10 miRNAs as endogenous controls

miRNA	mean *CT_0_*	stdev	geNorm
**191**	20.92	0.69	1.14
**744**	25.49	0.72	1.17
**152**	25	0.73	1.12
**MammU6**	17.12	0.75	1.22
**92a**	22.03	0.75	1.24
**29c**	26.15	0.78	1.26
**186**	23.69	0.78	1.17
**671-3p**	28.89	0.8	1.29
**26b**	23.75	0.8	1.19
**let-7d**	23.07	0.8	1.16

Mean *CT_0 _*calculations, standard deviations, and geNorm stability values resulting from using each of the 10 miRNAs with lowest standard deviation.

**Figure 8:**
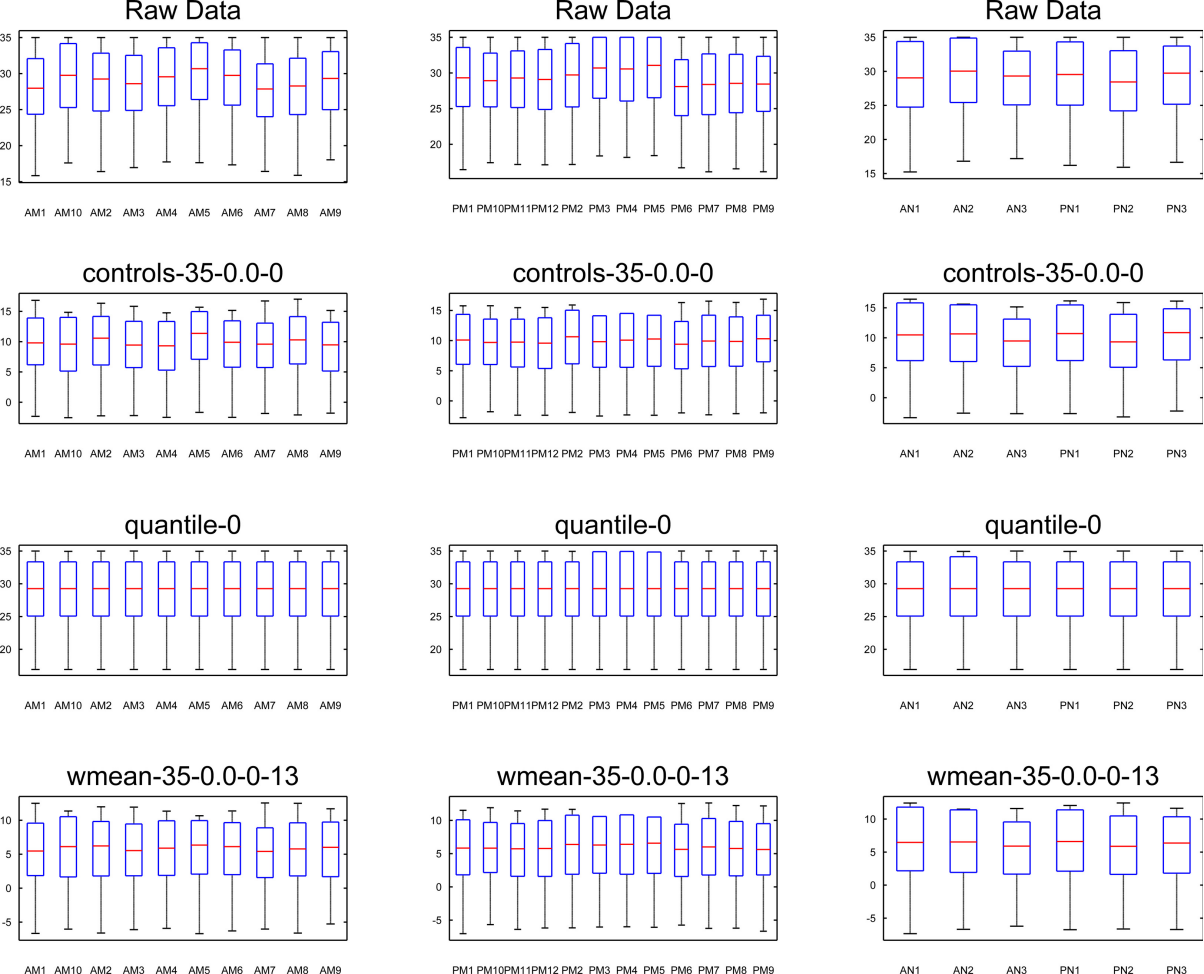
**Boxplots of raw and normalized data**. Here boxplots of the raw data in the first row, followed by boxplots of normalized data in each subsequent row are presented. The raw CT values are compared to the quantile normalized CT values and the ΔCT values produced by the endogenous controls and the weighted mean normalization with a weighted mean power of 13. The column on the left contains adult melanoma samples, the middle column contains pediatric melanoma samples, and the right column contains adult and pediatric nevus samples.

The proposed weighted mean normalization method could not be compared to quantile normalization in the same fashion as the other methods because quantile normalization does not have a value analogous to CT_0 _which could be evaluated for stability and compared to weighted mean normalization. However, the normalized data resulting from each method could be visualized and compared as boxplots. Figure 8 shows the distributions of the raw and normalized data. All normalization methods tend to decrease the variability compared to the raw data. Although quantile normalization forces all samples to have the same medians and distributions, Figure 8 shows that weighted mean normalization compares favorably with quantile normalization. Although the sample medians and sample standard deviations resulting from weighted mean normalization differ slightly, they are generally uniform, and they are clearly more uniform than the endogenous control normalization. Weighted mean normalization also tends to have lower standard deviation than quantile normalization. For three samples in the second group (PM3, PM4, and PM5) that have higher expression in the raw data, weighted mean normalization results in medians and standard deviations which are similar to the other samples, while quantile normalization seems to produce greater standard deviation for these samples.

**Figure 9:**
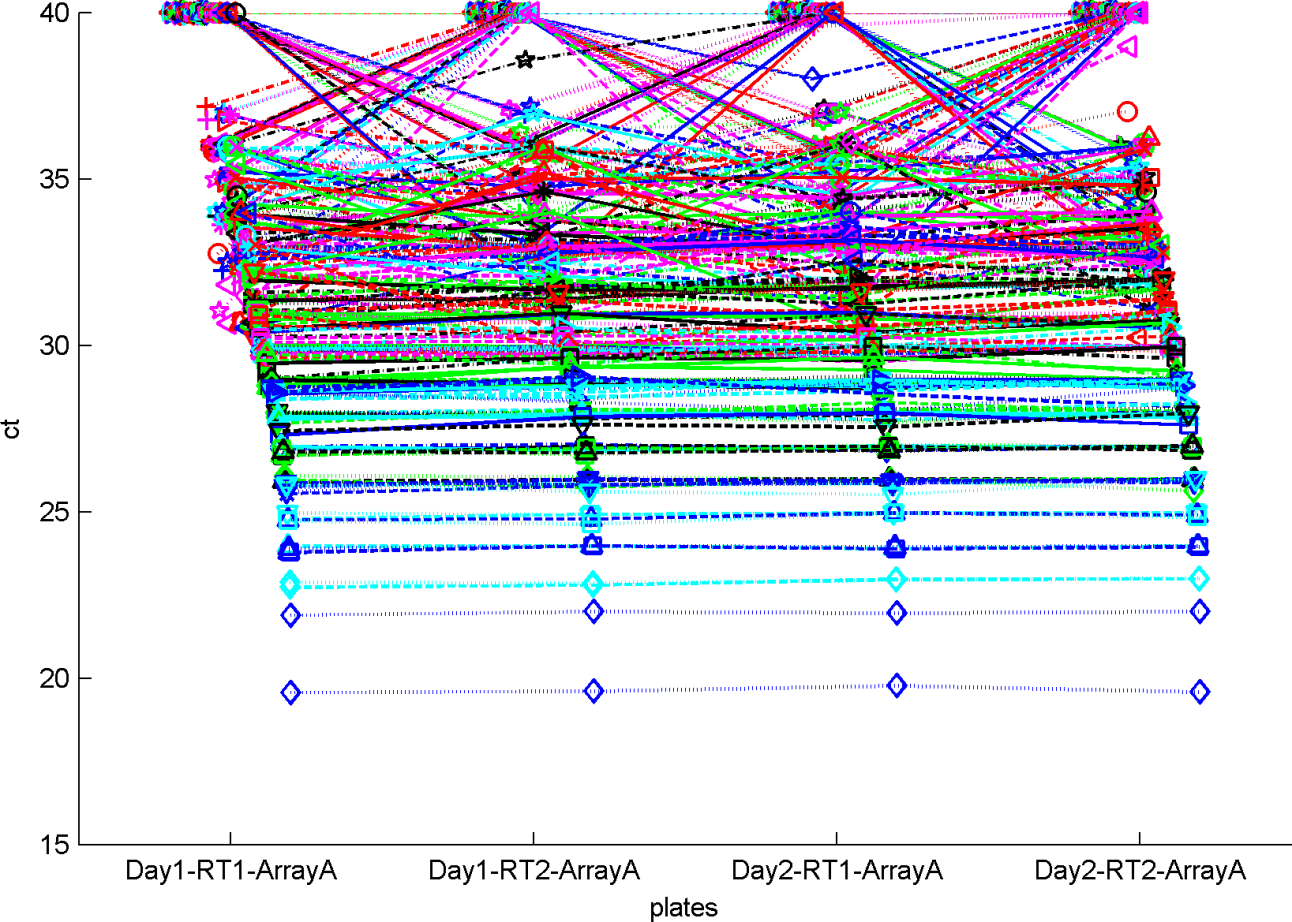
**Distribution of miRNA CT values on card A**. Each line represents the CT value of a particular miRNA across each of the four samples.

**Figure 10:**
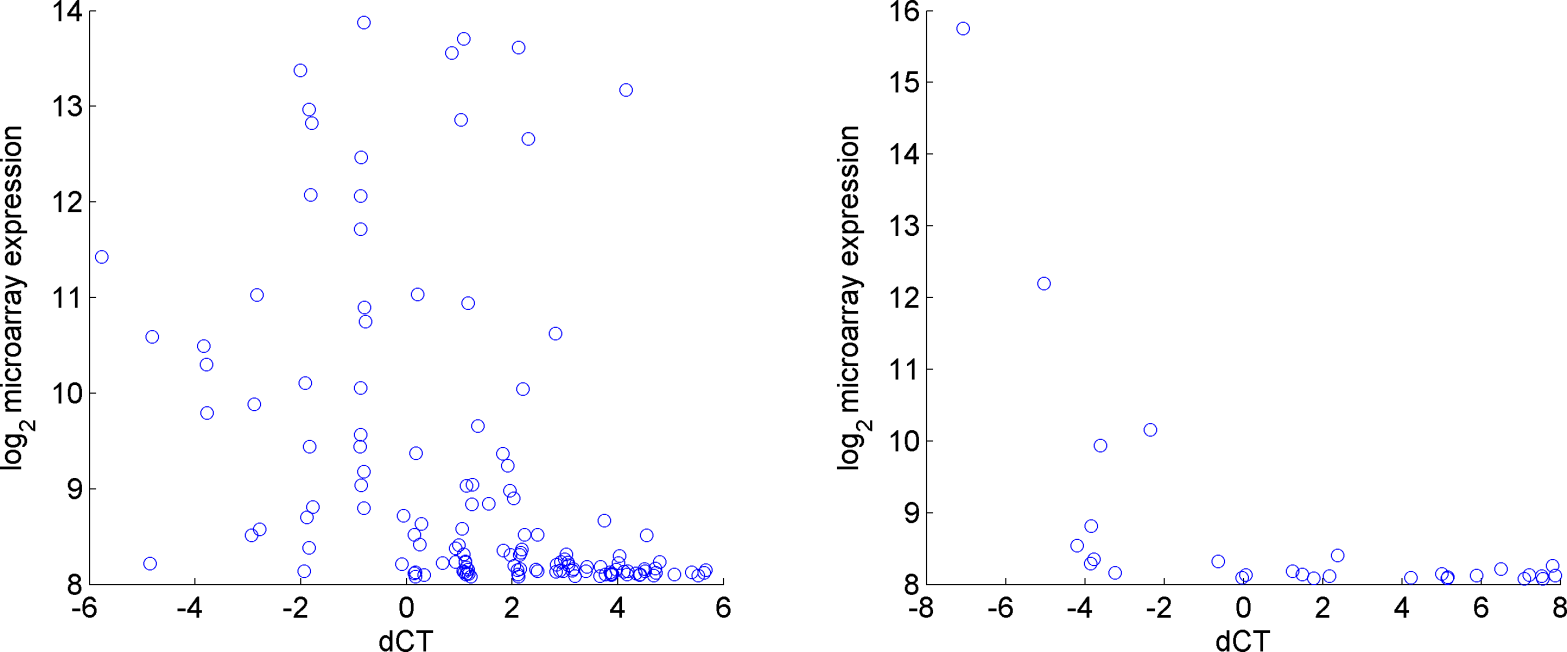
**RT-PCR expression vs. log microarray expression**. On the left each point represents the base 2 logarithm of the microarray expression vs. the ΔCT value for a particular miRNA for card A. On the right is the same plot for card B.

Having explored the problems of RT-PCR normalization, the consistency of miRNA expression experiments between RT-PCR and microarray technologies was of further interest to us. In order to explore this issue we used a dataset from Chen et al. [26], to explore the reproducibility of miRNA expression experiments across different platforms. Chen et al. explored the effect of different reverse transcription reactions as well as the effect of pre-amplification of a sample. They concluded that different reverse transcription reactions did not result in significant variation in CT values. Because the samples in their dataset were highly consistent, as shown in Figure 9, this dataset was not suitable for the evaluation of the different normalization methods presented in this work. We instead examined the effect of initial concentration of a miRNA on its correlation with the microarray experiment. Figure 10 contains a plot of the ΔCT value vs. the base 2 logarithm of the microarray expression for each miRNA for both cards in the experiment. The microarray data was log-transformed so that it would be on the same scale as the ΔCT values. We expect the ΔCT values to correlate negatively with the microarray values since lower ΔCT values indicate higher expression, and Figure 10 shows that generally lower ΔCT values are associated with higher microarray expression.

**Figure 11:**
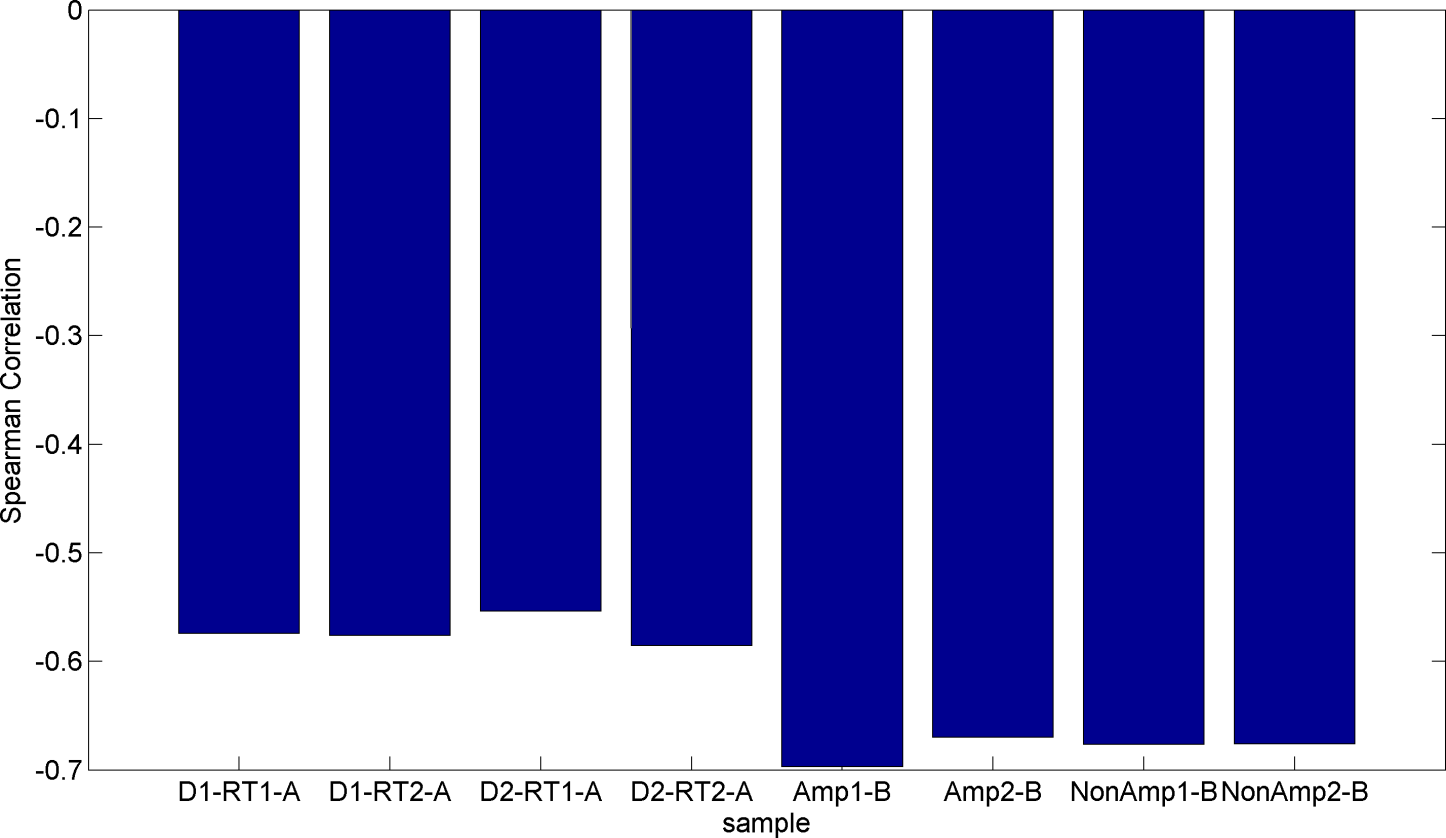
**Spearman correlation of each RT-PCR sample with microarray expression**. Here we show the Spearman correlation of the ΔCT values with the base 2 logarithm of microarray expression for each RT-PCR sample. The left four samples come from card A and consist of two different reverse transcription reactions each performed on one of two different days. The right four samples come from card B and consist of two pre-amplified and two non-amplified RT-PCR samples.

**Figure 12:**
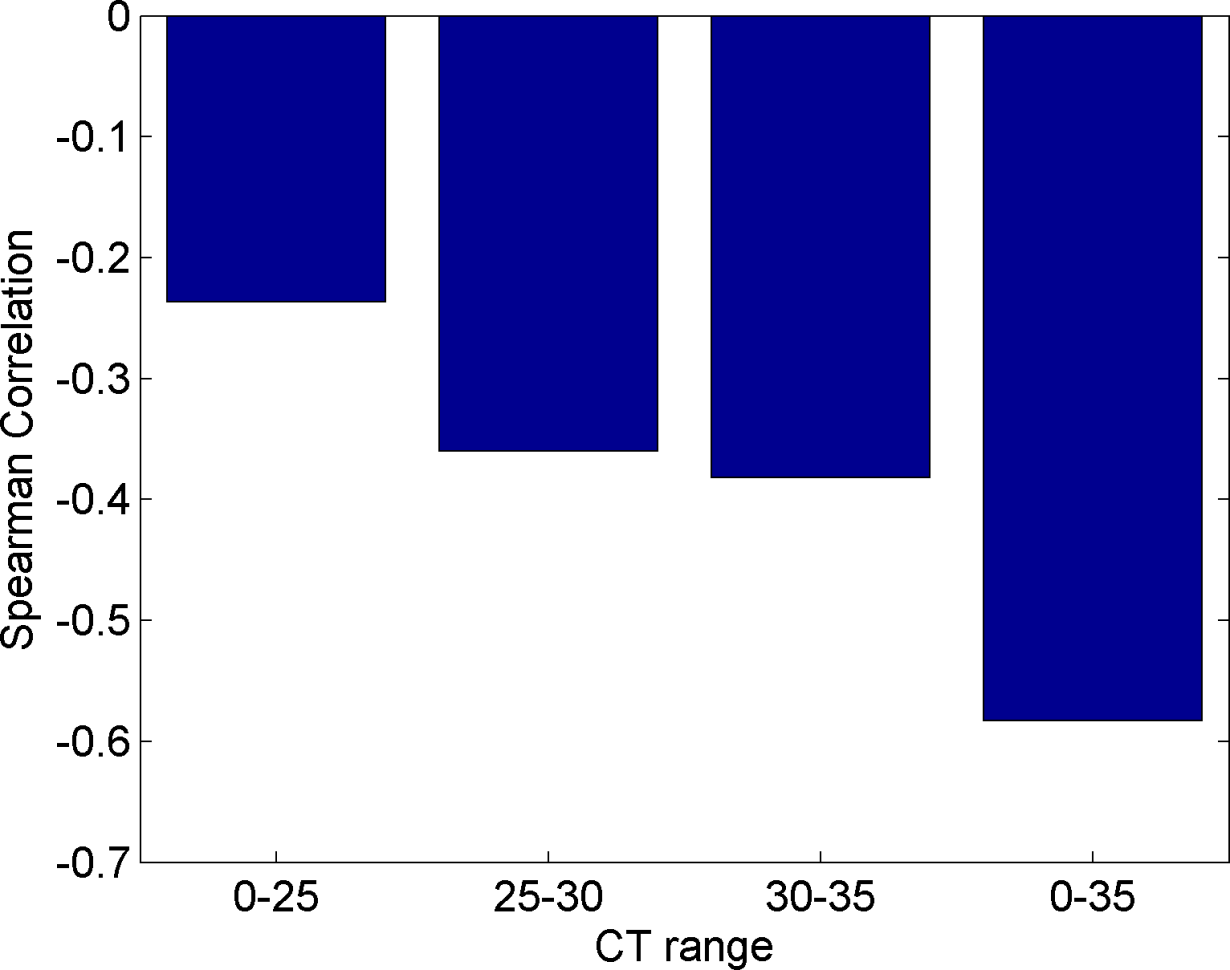
**Correlation of card A by range of CT values**. Here we divided the miRNAs detected on both card A and the microarray into ranges of CT values and calculated the Spearman correlation of the miRNAs' ΔCT values with the base 2 logarithm of their microarray expression.

**Figure 13:**
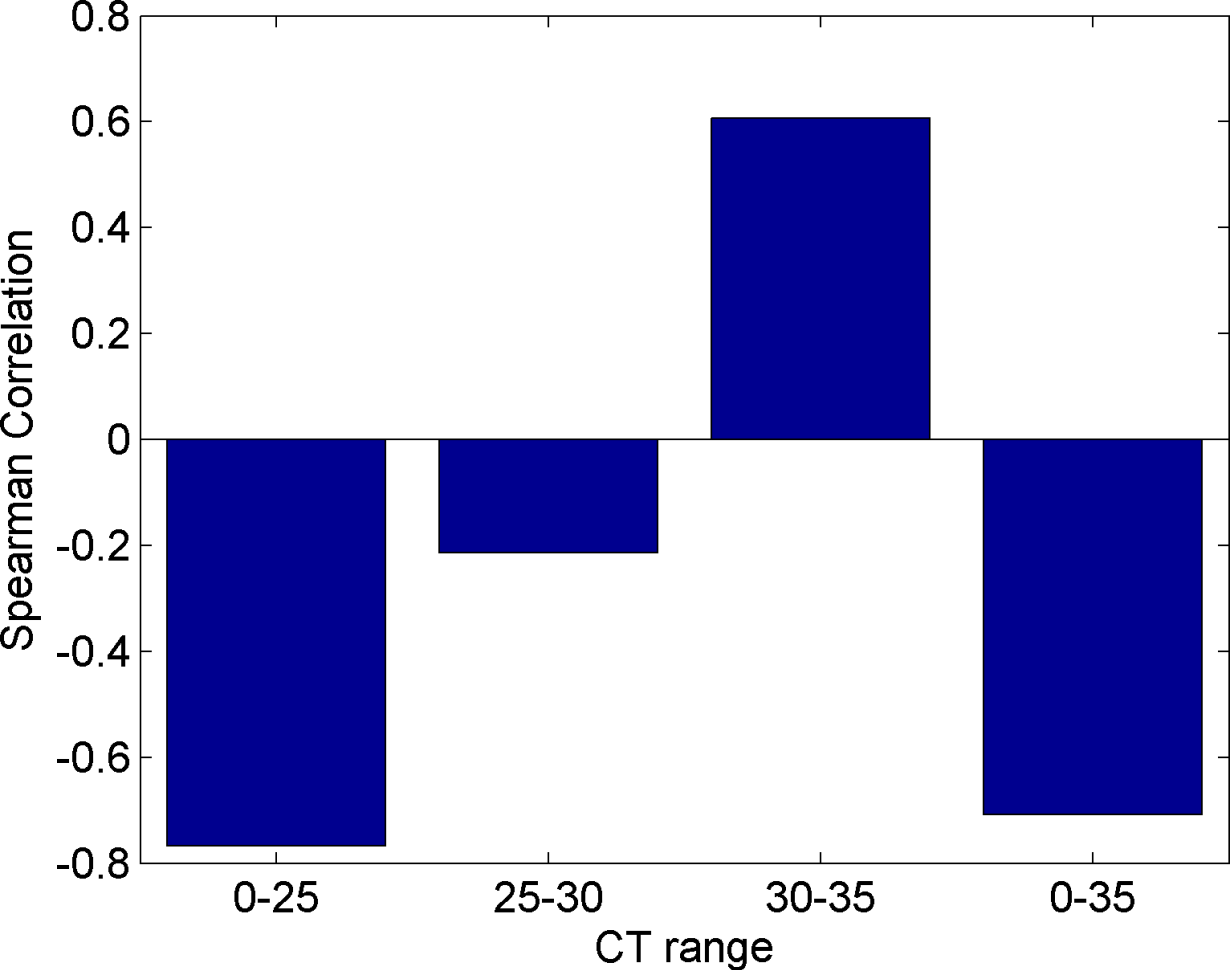
**Correlation of card B by range of CT values**. Here we divided the miRNAs detected on both card B and the microarray into ranges of CT values and calculated the Spearman correlation of the miRNAs' ΔCT values with the base 2 logarithm of their microarray expression.

Figure 11 displays the Spearman correlation of each of the RT-PCR samples with the microarray data. The experiment used two different arrays or cards for RT-PCR. The card "A" contains well-characterized miRNAs, while card "B" contains newly discovered, less well-known miRNAs [26]. We observe that card B is more highly correlated than card A. However, card A had 134 miRNAs detected that were also present on the microarray while card B only detected 29 miRNAs that were present on the microarray. Although the values are similar, the pre-amplified samples have a higher correlation with the microarray than the non-amplified samples. Due to the bias described previously we expected to observe miRNAs with higher initial concentrations (lower CT values) to correlate better with the microarray. In order to test this hypothesis we partitioned the data from each card into bins based on CT ranges. In card "A", contrary to our expectation, we observe that the Spearman correlation improves slightly for higher CT values as shown in Figure 12. We also observe that using all miRNAs results in a stronger correlation than any individual range of CT values. In Card "B," shown in Figure 13, we do observe stronger correlation values with lower CT ranges. The highest bin, containing miRNAs with average CT values ranging from 30 to 35 contradicts the expectation of negative correlation, and we observe a large positive correlation, however this correlation is not significant, indicating that the measurement of the expression of the specific miRNAs that fell in this range exhibited considerable variability between RT-PCR and microarray data.

## Conclusions

We explored the phenomenon whereby differences in the initial sample size of miRNA in an RT-PCR experiment were magnified with increasing CT levels. This was illustrated by the strong correlation of the CT values of the individual miRNAs with the average CT values of all miRNAs and by the increased sensitivity in the CT values of the low-abundant miRNAs to the average CT values. We conclude that a systematic bias in RT-PCR exists in which the fluctuations in the CT are dependent on the expression levels of the particular miRNAs. We further proposed a novel data-driven method of addressing this bias by using the weighted mean instead of an endogenous control in the calculation of ΔCT. We demonstrated that the new normalization method produces lower standard deviations and is more stable than other methods.

Note that, while the power parameter in the weighted mean normalization method provides a convenient way of adjusting how much one wishes to let the less stable microRNAs influence the normalization of other microRNAs, its optimization currently requires enumeration of different values and using the one with the best overall stability. Several CT_0 _values can be calculated for different values for the weighted mean power, subsequently the value of the power that produces the lowest standard deviation or is determined to be the most stable by geNorm can be used for normalization. The standard deviation or geNorm stability calculations are two methods to quantitatively determine the ideal weighted mean power. Other criteria, such as significance of the differentially expressed microRNAs can be utilized in this optimization. Furthermore, a different custom *CT_0 _*value for each microRNA may be used, such that each microRNA is normalized differently, dependent on its average expression level.

We further examined the reproducibility of miRNA expression experiments across two different platforms by comparing RT-PCR and microarray results. We explored the relationship between the CT value and the consistency of the expression of a miRNA between RT-PCR and microarray. We leave as a future work the comparison of the ability of different normalization methods to detect differentially expressed genes.

## Competing interests

The authors declare that they have no competing interests.

## Authors' contributions

AS conceived of the study and coordinated the project. RQ implemented the method and performed the experiments. RQ contributed to the design and testing of the method. All authors participated in the analysis of the results. RQ and AS contributed to the writing of the manuscript. All authors read and approved of the final draft.
